# Heterotopic ossification in the post cruciate ligament of the knee: a case report and literature review

**DOI:** 10.1186/s12891-021-04176-x

**Published:** 2021-03-26

**Authors:** Cun Li, Zonggui Huang, K. C. Anil, Chendeng Lao, Qianghua Wu, Hongmian Jiang

**Affiliations:** 1 Department of Sports Orthopaedics, The First People’s Hospital of Nanning, Nanning, People’s Republic of China; 2 Department of Orthopaedics, The First Affiliated Hospital of Guangxi Medical University, Nanning, People’s Republic of China; 3 Department of Pathology, The First People’s Hospital of Nanning, Nanning, People’s Republic of China

**Keywords:** Heterotopic ossification, Posterior cruciate ligament, Arthroscopy, Case report

## Abstract

**Background:**

Heterotopic ossification (HO) is noted most frequently in periarticular muscles and has not yet been reported in the cruciate ligaments of the knee. We present a rare case of symptomatic ossification of the posterior cruciate ligament (PCL).

**Case presentation:**

A 59-year-old woman had a 2-year history of knee pain that was getting worse during knee motion and had restricted knee motion for 1 year. X-rays could not show the lesion clearly. Multi-planar computed tomography demonstrated ossification within the PCL with mild osteoarthritic changes and excluded any other intra-articular pathology. The patient underwent arthroscopic debridement and then experienced immediate relief of pain and complete recovery of range of motion.

**Conclusion:**

This is the first report of HO in the PCL as a possible cause of knee pain and restricted knee motion. On the basis of literature review, this case elaborates the difference between HO and calcification in the ligaments, the related factors inducing HO and the undefined pathogenesis, and favorable prognosis after adequate treatment.

## Background

Heterotopic ossification (HO) refers to the pathologic formation of new bone in non-ossified tissues. Different from metastatic calcification and dystrophic calcification, HO commonly occurs after injuries and surgery and forms mature lamellar bone with limited capacity for growth [1]. HO commonly occurs in the skeletal muscle, subcutaneous tissue, skin, and fibrous tissue adjacent to joints and is noted most frequently in periarticular muscles, with decreasing frequencies in the hip, elbow, knee, and shoulder [2, 3]. The prevalence of HO is as high as 90% after hip arthroplasty and acetabular fracture, 40% after elbow fracture, and 25% after knee dislocation [3, 4]. However, this pathologic process has not yet been reported in the cruciate ligaments of the knee. Here, a case report of a patient with knee pain and restricted knee motion is presented, which was diagnosed as HO of the posterior cruciate ligament.

## Case presentation

A 59-year-old female presented to our outpatient clinic complaining of pain in the left knee, with decreased range of motion (ROM) for 1 year. When she visited our inpatient department on the next day, her ROM of the left knee was restricted from 0° to 15°. She had a history of left knee pain in the past 2 years with the absence of trauma. The pain was non-specific and significantly exacerbated by knee activity. She had no prior consultation for her symptoms but occasionally took diclofenac sodium capsules for pain relief. The pain became so severe that she could no longer walk. Her past history of illness revealed that she had atrial fibrillation and a 1-week history of swelling and pain in both legs 3 years ago. Therefore, she underwent ultrasound examination and was diagnosed with multiple thrombosis in the bilateral lower limb arteries, which was more serious in the right lower limb with complete thrombus occlusion occurring in the right popliteal artery. She underwent interventional right iliac artery thrombectomy with stent implantation and balloon dilatation of the right anterior tibial artery in our hospital 3 years ago. Since then, she has been taking anticoagulants (warfarin) for atrial fibrillation until she presented at our department. The patient had no history of endocrine and metabolic disorders such as diabetes, hyperthyroidism, and gout. During physical examination, obvious and unbearable pain upon movement was noted without any knee swelling and tenderness. Signs related to meniscus pathology or joint laxity were not found.

### Investigations

**Fig. 1:**
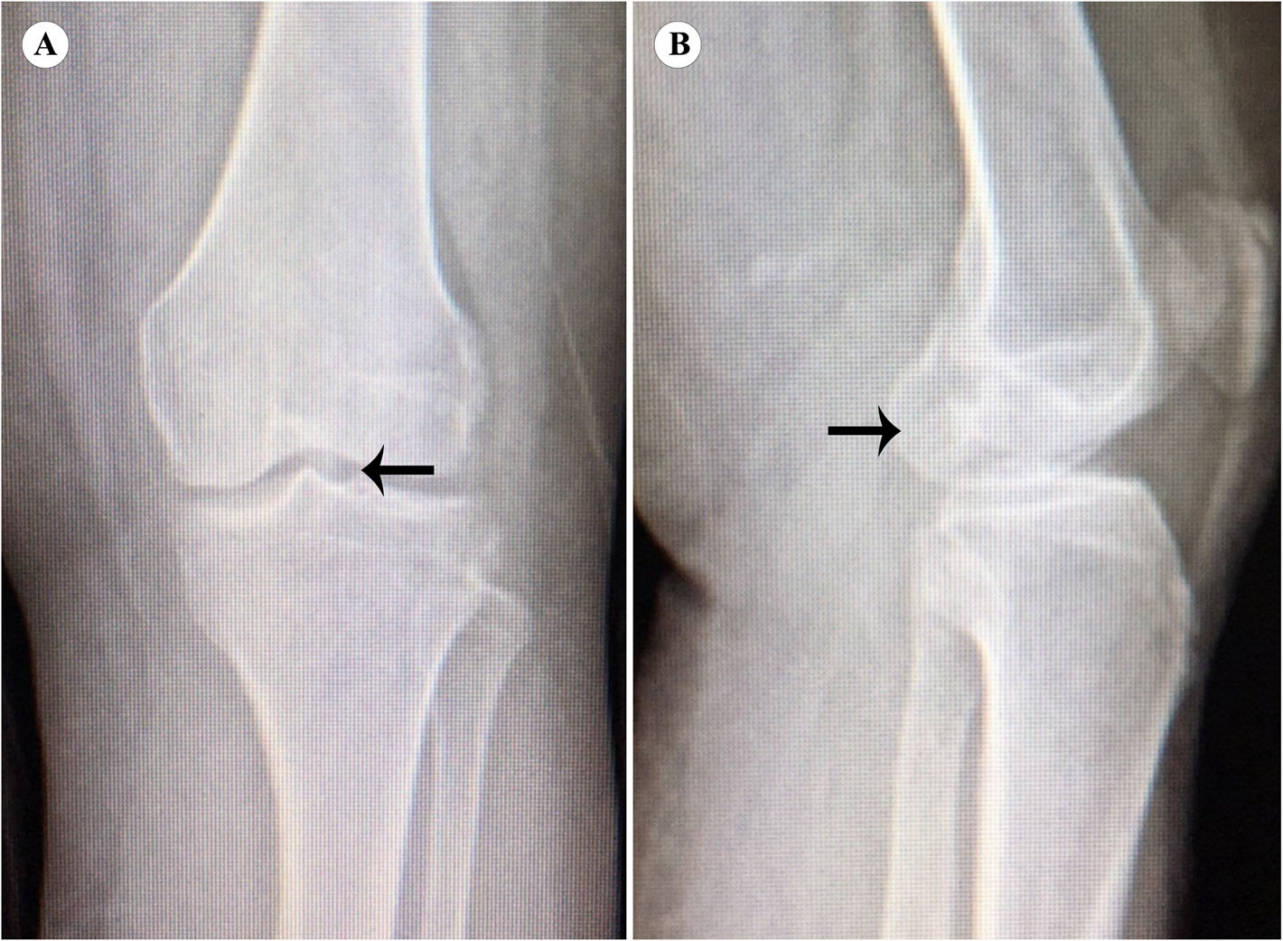
A dimly glimpsed high-density mass within the intercondylar notch is showed on X-ray of the left knee (arrow)

**Fig. 2:**
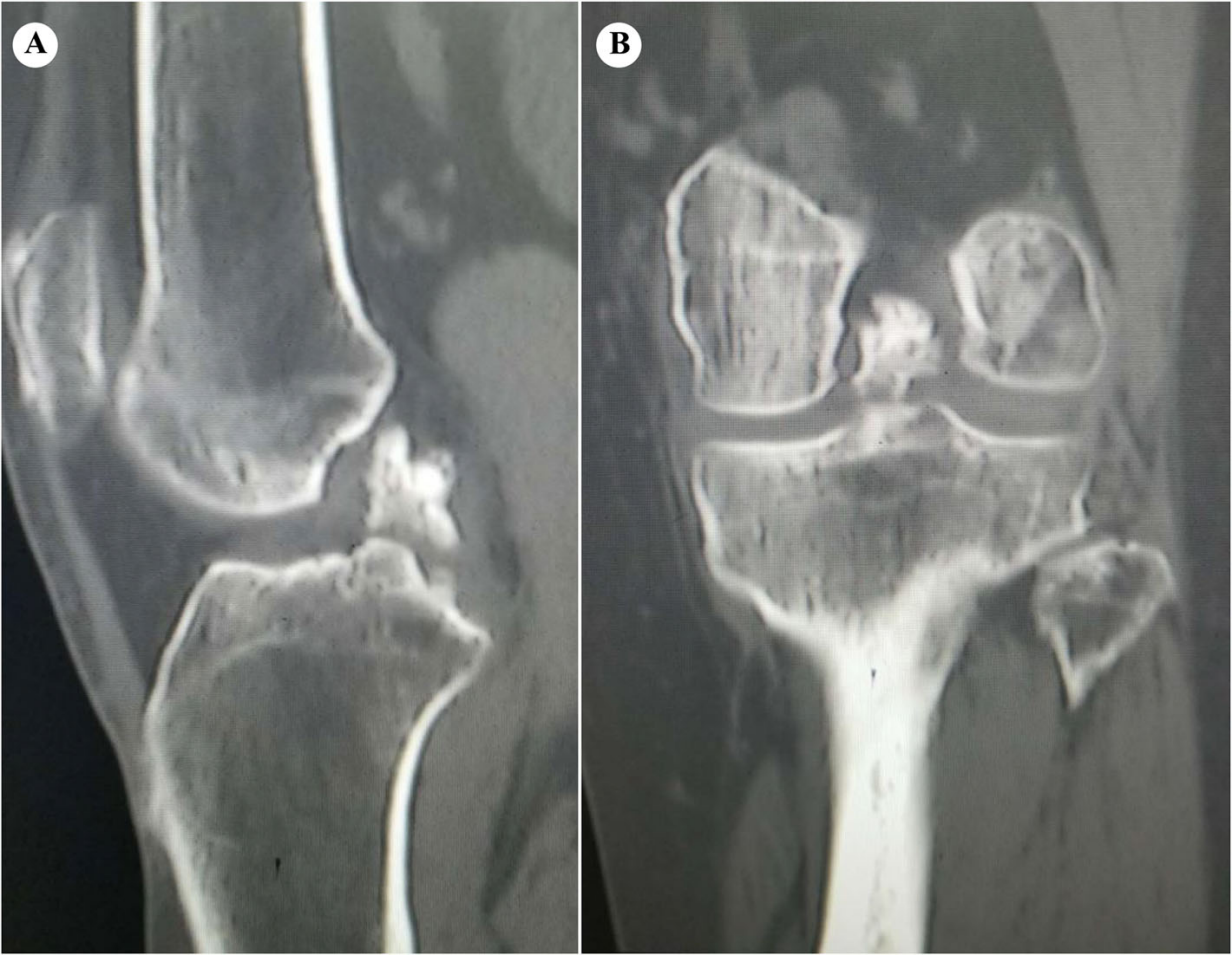
High-density mass is seen in the PCL by multi-planar reconstruction CT

Blood tests were performed while the patient was hospitalized. The results revealed normal range of calcium and glucose at 2.35 and 4.99 mmol/L, respectively; however, the patient’s blood uric acid was high (480 μmol/L). The patient had normal hemoglobin of approximately 130 g/L. Three days after arthroscopic debridement, the patient developed mild leukocytosis, which was believed to be a response to surgical stress. The results of biochemical and hematologic tests were within the normal range. Anteroposterior and lateral radiographs of the left knee demonstrated a high-density shadow within the intercondylar notch with mild osteoarthritic changes (Fig. 1). The high-density mass on plain radiographs was so inconspicuous that our radiologist did not notice or report it. Multi-planar computed tomography (CT) positioned the lesion precisely and excluded any other intra-articular pathology that could cause limitation of joint movement. The mass appeared as a non-structural lesion in the intercondylar area by coronal CT (Fig. 2a). The sagittal CT image revealed the mass inside the PCL (Fig. 2b). CT showed the mass with heterogeneous density. In both X-ray and CT images, the mass was shown as high density, which made it difficult to determine whether it was an ossified or a calcified lesion. Although magnetic resonance imaging (MRI) is meaningful and necessary for a full evaluation of the ligaments in our patient, it was not recommended because of the presence of a stent of unknown material in the body.

### Treatment

**Fig. 3:**
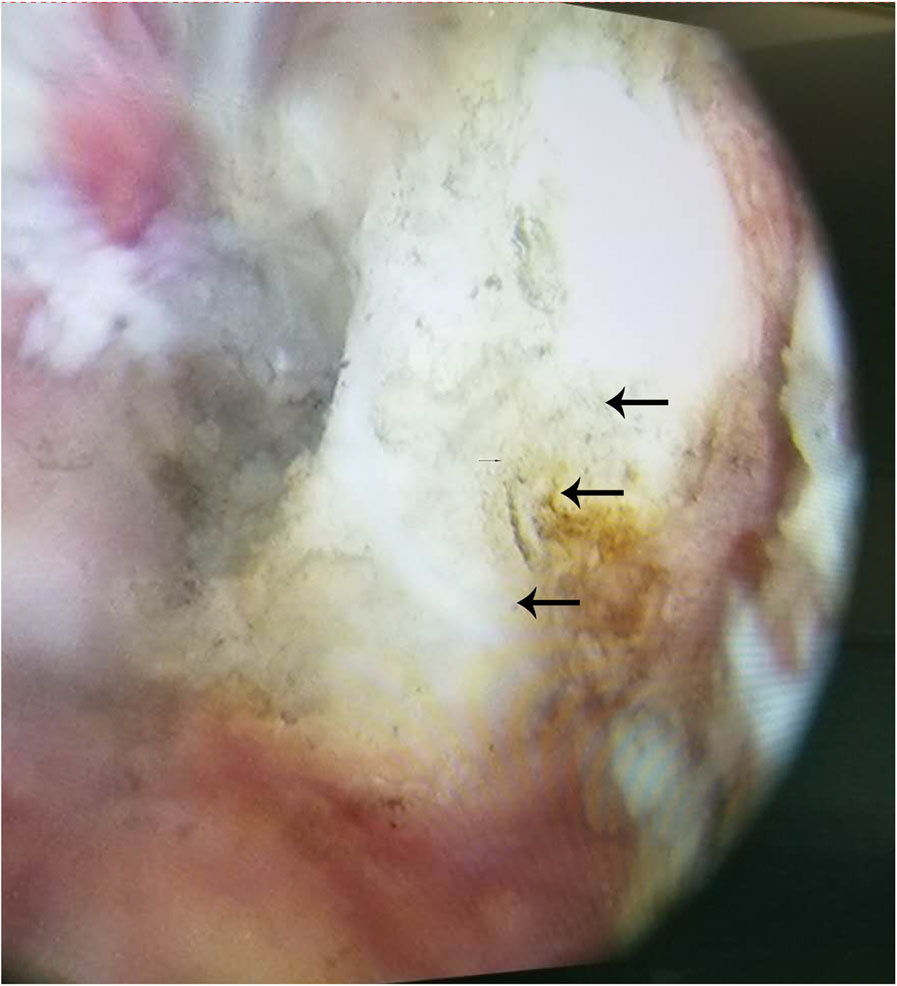
Arthroscopic view of local ossification of the PCL (arrows)

Because we speculated that the patient’s discomfort resulted from mechanical locking and entrapment by the dense mass, we recommended surgery and performed arthroscopy accordingly. Intraoperatively, a hump of the PCL was revealed by arthroscopic examination, while other pathological changes were not found. The fiber fascicles of the PCL appeared after debridement of the synovial membrane covering the pathological site of the PCL. We slit open the fascicles and exposed the mass, which appeared grayish-white and seemed to be hard based on the macroscopic appearance (Fig. 3). The mass was so closely adhered to the fibrous bundles of the ligament that debridement of the lesion was carefully performed. The pathological tissue obtained from surgery was sent for histopathology.

**Fig. 4:**
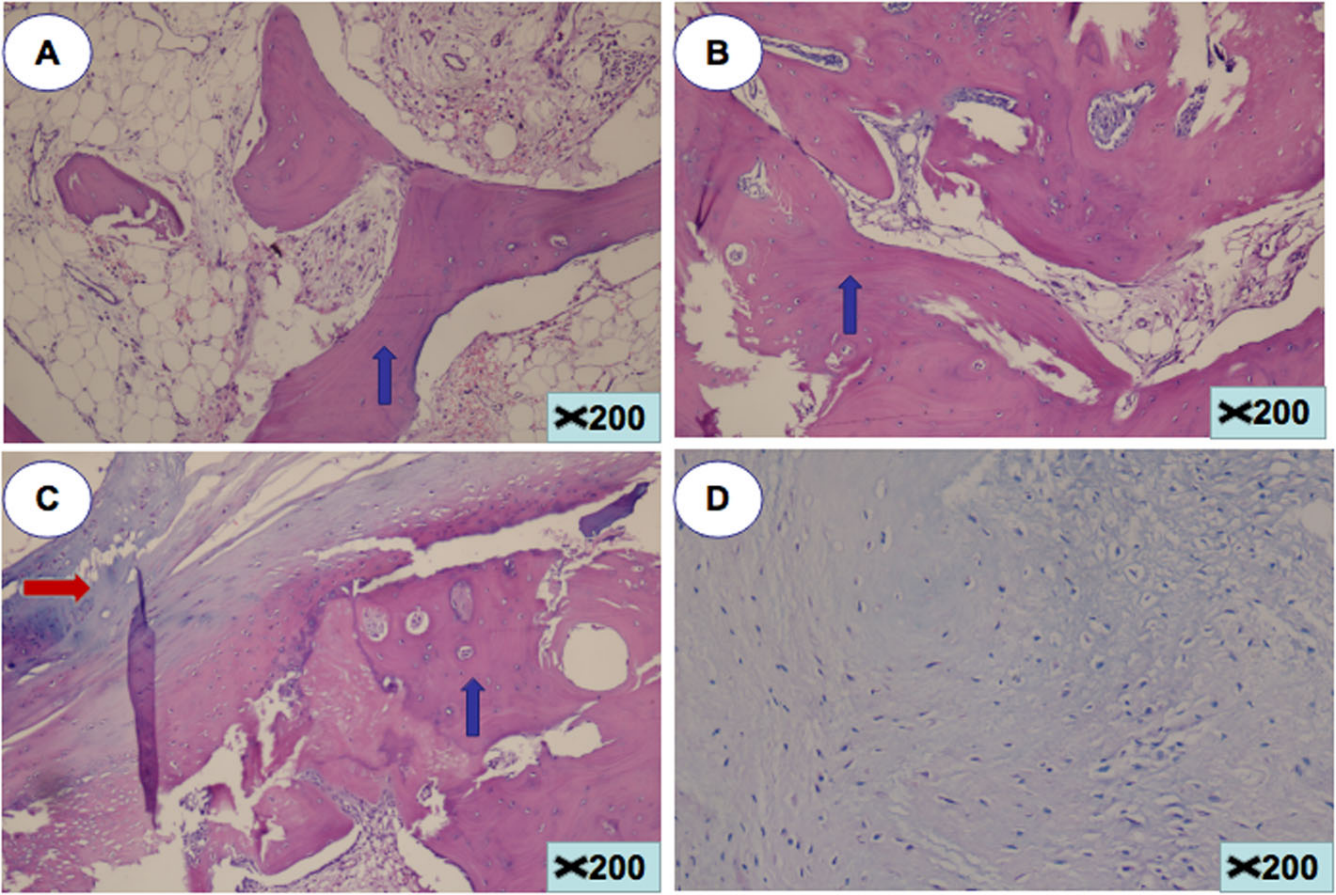
Histopathologic evidence of heterotopic ossification (hematoxylin and eosin, original magnification× 200). **a**, **b** Bone staining clearly red (blue arrow). **c** Cartilage staining light blue (red arrow) and bone (blue arrow). **d** Chondroblasts proliferating

Pathologically, the tissues were calcified mature lamellar bone with completely fibrotic bone marrow, which was infiltrated by a few chronic inflammatory cells (Fig. 4). The pathological tissue obtained from surgery was stained with hematoxylin–eosin. The finding of osteogenic components or lamellar bone and chondrogenic components or cartilage led to the diagnosis of HO. The hallmark of osteogenic components, which were stained clearly red, is lamellar characteristics. The chondrogenic components were stained light blue with a few chondroblasts.

### Outcome and follow-up

Following the surgery, the patient verbalized complete relief of her symptoms. Moreover, she was allowed full ROM and weight bearing with no assistance. No complications were observed during the postoperative course. The patient was discharged after wound healing 2 weeks later. At 1-year follow-up, clinical examination demonstrated no signs of recurrence, and the patient gained full ROM with no pain or instability.

## Discussion and conclusions

To our knowledge, this is the first case of isolated PCL ossification reported in the literature. Two studies reported the occurrence of an ossicle within the substance of the anterior cruciate ligament (ACL); unfortunately, both of them considered that it was a variation of the ACL [5, 6]. Jung described posterolateral capsular heterotopic bone formation with decreased ROM of the knee, which needed surgical excision after PCL reconstruction [7]. In this study, only radiographs were used to diagnose HO without pathological findings, which were deficient or inadequate in terms of the methodology used to diagnose HO. Unlike cases of calcification, cases involving isolated symptomatic ossification of a ligament within the knee have been rarely reported [8–12]. Although their clinical manifestations and treatment methods are largely similar, the pathologies are completely different. Typical clinical findings such as restricted ROM and knee pain with or without a history of major trauma are common in ossification and calcification of a ligament [8–12].

Plain radiographs are primarily used to detect HO, but they can lead to misdiagnosis such as loose bodies [13]. CT is often used for the early evaluation of the extremity and demonstrates calcium within the ligament with high Hounsfield units (> 200). However, the density of ligament calcification is more uniform than that of ligament ossification, which is often accompanied by a shadow with slightly lower density in the center of the lesion. Although MRI was not performed in our case, some studies showed that MRI has advantages in displaying the specific location and identifying the nature of the mass [13–15]. MRI not only assesses the relation with lesion and peripheral structures, but also shows the isointensity to the normal bone marrow with a hypointense rim [14], which is helpful to differentiate it from calcified lesions. In addition, MRI can display abnormalities of other intra-articular structures, such as ligamentous and meniscal tears.

The difference between ligament ossification and calcification can be elaborated on the basis of the pathological findings. Pathologically, ligament calcification, which resembles calcific tendinitis of the rotator cuff, appears as a simple deposition of calcium salts, and inflammatory cells occasionally infiltrate the ligament [8–10]. However, the pathological material of our case consists of a mature lamellar bone with a central bone marrow resembling that acquired from HO [16].

HO has been increasingly recognized as a complication following major orthopedic surgeries, spinal cord injury, traumatic brain injury, blast injury, elbow and acetabular fractures, and thermal injury [2, 3]. Chantraine proposed that repeated microtrauma at the site of the joints and vascular disorders such as venous stasis and arteriovenous shunting, which cause metabolic changes that lead to cell differentiation, might play a major role in HO metaplasia [1]. Our case had no obvious history of trauma and relevant family history. We speculated that her medical history of artery thrombosis or repeated minor injuries that went unnoticed contributed to her HO. Nevertheless, the related factors inducing the disease cannot be verified.

Although current research on ligament ossification seeks to better understand the underlying cellular, biochemical, and mechanical processes, its pathogenesis remains unclear. One predominant theory has emerged to explain its occurrence [16–18]. Chalmers proposed a pathogenesis on the basis of three essential elements: an inducing agent, progenitor cells, and an environment conducive to bone production [17]. This concept is currently widely accepted. On the basis of Chalmers’ research, Kaplan suggested that progenitor cells are ubiquitous to various tissues, including ligament, muscle, and vessels. As a result of injury or other causes, the local inflammatory environment may result in the differentiation of these cells into osteoprogenitor cells capable of forming HO and is affected by oxygen tension, pH, availability of micronutrients, and mechanical stimuli [18]. To characterize the properties of cells derived from tissue containing pre- and mature ectopic bone, Benjamin studied several mouse models of HO, including two models of trauma-induced HO [19]. They noted that cells isolated from trauma sites in two distinct models exhibited increased proliferation and osteogenic differentiation compared with cells isolated from uninjured controls [19]. That is, trauma as an inducer enhances the proliferation and osteogenic differentiation properties of cells at the injured site. Although our case had no history of injury, the metabolic changes resulting from vascular disorders or unnoticed minor injuries, which led to local necrosis or degeneration, probably created an inflammatory environment that induced the occurrence of HO.

In previous studies, conservative treatments such as oral nonsteroidal anti-inflammatory drugs, diphosphonates, and low-dose local radiotherapy have been used to treat HO [18, 20, 21]. However, we could not find any literature report about ossification within the PCL. Several studies have reported the use of arthroscopic debridement operation in the treatment of calcification lesions within the cruciate ligament [8–10]. Likewise, the patient in the present case was treated with arthroscopic debridement of the lesion within the PCL. Although the ossified tissue could not be completely removed due to the close adhesion between HO and ligament fibers, we removed the lesion as much as possible to maintain the integrity of the PCL intraoperatively. The patient’s pain and restricted ROM subsided immediately after the surgery.

Arthroscopic debridement of the lesion can directly eliminate the symptoms of entrapment and mechanical locking, which cannot be achieved by conservative treatments. Our findings suggest that the arthroscopic removal of the lesion is an effective and necessary procedure for treating ossification of the PCL, especially when the symptoms are considered to be primarily caused by entrapment and mechanical locking.

In conclusion, HO in the PCL is rare. This is the first report of HO in the PCL as a possible cause of knee pain and restricted knee motion. HO is often easily misdiagnosed as calcification of the ligament. CT scan and MRI are important in differentiating between ossification and calcification; however, histopathological examination can confirm the bone nature of the lesion, which would make a definitive diagnosis. Arthroscopic debridement of the lesion is an effective and necessary procedure for treating ossification of the PCL.

## Data Availability

All the data needed to achieve the conclusion are presented in the pater.

## Abbreviations

HO: Heterotopic ossification
PCL: Posterior cruciate ligament
CT: Computerized tomography
MRI: Magnetic resonance imaging
ROM: Range of motion
ACL: Anterior cruciate ligament
